# Fructan biosynthesis and degradation as part of plant metabolism controlling sugar fluxes during durum wheat kernel maturation

**DOI:** 10.3389/fpls.2015.00089

**Published:** 2015-02-20

**Authors:** Sara Cimini, Vittoria Locato, Rudy Vergauwen, Annalisa Paradiso, Cristina Cecchini, Liesbeth Vandenpoel, Joran Verspreet, Christophe M. Courtin, Maria Grazia D'Egidio, Wim Van den Ende, Laura De Gara

**Affiliations:** ^1^Laboratory of Plant Biochemistry and Food Sciences, Campus Bio-Medico UniversityRome, Italy; ^2^Laboratory for Molecular Plant Biology and Leuven Food Science and Nutrition Research Centre (LFoRCe), KU LeuvenLeuven, Belgium; ^3^Dipartimento di Biologia, Università degli Studi di BariBari, Italy; ^4^Consiglio per la Ricerca e la Sperimentazione in Agricoltura, Unità di ricerca per la Valorizzazione Qualitativa dei CerealiRome, Italy; ^5^Laboratory of Food Chemistry and Biochemistry, KU LeuvenLeuven, Belgium

**Keywords:** durum wheat, fructosyltransferase, fructan exohydrolase, kernel development, bio-active molecule

## Abstract

Wheat kernels contain fructans, fructose based oligosaccharides with prebiotic properties, in levels between 2 and 35 weight % depending on the developmental stage of the kernel. To improve knowledge on the metabolic pathways leading to fructan storage and degradation, carbohydrate fluxes occurring during durum wheat kernel development were analyzed. Kernels were collected at various developmental stages and quali-quantitative analysis of carbohydrates (mono- and di-saccharides, fructans, starch) was performed, alongside analysis of the activities and gene expression of the enzymes involved in their biosynthesis and hydrolysis. High resolution HPAEC-PAD of fructan contained in durum wheat kernels revealed that fructan content is higher at the beginning of kernel development, when fructans with higher DP, such as bifurcose and 1,1-nystose, were mainly found. The changes in fructan pool observed during kernel maturation might be part of the signaling pathways influencing carbohydrate metabolism and storage in wheat kernels during development. During the first developmental stages fructan accumulation may contribute to make kernels more effective Suc sinks and to participate in osmotic regulation while the observed decrease in their content may mark the transition to later developmental stages, transition that is also orchestrated by changes in redox balance.

## Introduction

Cereals are basic components of the human diet. Wheat is one of the primary grains consumed by humans, with about 700 million tons being annually harvested (Charmet, 2011). Interest in cereals as a source of bioactive and functional molecules has increased. The enrichment of pasta and other cereal-derived foods with immature kernels is an interesting prospect in the field of functional foods (Paradiso et al., 2006; Casiraghi et al., 2013).

Kernel maturation is a complex process controlled by several factors, both of endogenous and exogenous origin (hormones, photosynthetic efficiency, macro- and micronutrient availability, pests, etc.) (Sabelli and Larkins, 2009). The various stages of kernel maturation show almost the same trend over time, irrespective of the variable climatic conditions and the geographical areas of cultivation (Simmonds and O'Brien, 1981).

After fertilization, cell proliferation starts in the endosperm leading to the formation of a multinucleated syncytial tissue [1–5 days after anthesis (DAA); (Olsen, 2001)]. Afterwards, a cellularization process occurs, followed by a period of grain filling during which the water content increases (around 6–24 DAA; Altenbach et al., 2003). During this stage, cell division ends and cell enlargement begins in order to facilitate the accumulation of reserves. Recent transcriptomic studies on wheat caryopses indicate that the main reprogramming point of gene expression occurs during the transition from cell division to the grain-filling stage (7–14 DAA; Laudencia-Chingcuanco et al., 2007; Wan et al., 2008). At the beginning of grain development (1–7 DAA), the expression of genes involved in cell division, nucleic acids and protein metabolism and photosynthesis are observed. These genes show their maximal expression levels at 7 DAA, after which they are strongly down-regulated (Laudencia-Chingcuanco et al., 2007; Wan et al., 2008).

Wheat endosperm accumulates predominantly starch, storage proteins and lipids (Altenbach et al., 2003). Genes associated with starch and protein metabolisms usually have a bell-shaped expression pattern with a maximum at around 14 DAA. On the other hand, storage proteins and defense protein transcripts generally reach their maximum level during grain filling (around 21 DAA) and tend to be maintained until the end of maturation (Laudencia-Chingcuanco et al., 2007; Wan et al., 2008).

At about 25–28 DAA, wheat caryopses growing in temperate climates enter the last period of maturation, accompanied by major water losses. The acquisition of desiccation tolerance is crucial to allow the embryo to pass from the quiescent period to germination under suitable conditions (Angelovici et al., 2010). When wheat caryopses reach physiological maturity, the endosperm cells undergo programmed cell death (Olsen et al., 1999; Young and Gallie, 2000; Olsen, 2001). This phase is accompanied by remarkable shift of redox pairs toward the oxidized forms (De Gara et al., 2003). The timing of programmed cell death, with the consequent end of the storing process, has been suggested to be also controlled by the alteration in ascorbate level and metabolism occurring at this stage (Paradiso et al., 2012).

Regulated changes in storage reserves have been observed during wheat kernel development and mainly concern soluble and insoluble carbohydrates as well as storage proteins. Starch accumulation is mainly responsible for grain size and yield, accounting for 60–75% of the dry matter (dm) at the end of maturation, while soluble sugars such as glucose (Glc) and fructose (Fru) are present at this stage in very low amounts (Rahman et al., 2000; Slattery et al., 2000; De Gara et al., 2003).

A class of soluble Fru based oligo- and poly-saccharides, named fructans, are known to have positive effects on human health (Van den Ende et al., 2011a) and may fulfill a physiological role during wheat kernel development that requires further investigation. A study performed on 45 cultivars of durum wheat indicated that a substantial amount of fructans (up to 25% of dm) is present at 15 DAA (Paradiso et al., 2008) while, in mature durum wheat kernels, fructan content is about 2% of dm (De Gara et al., 2003).

**Figure 1:**
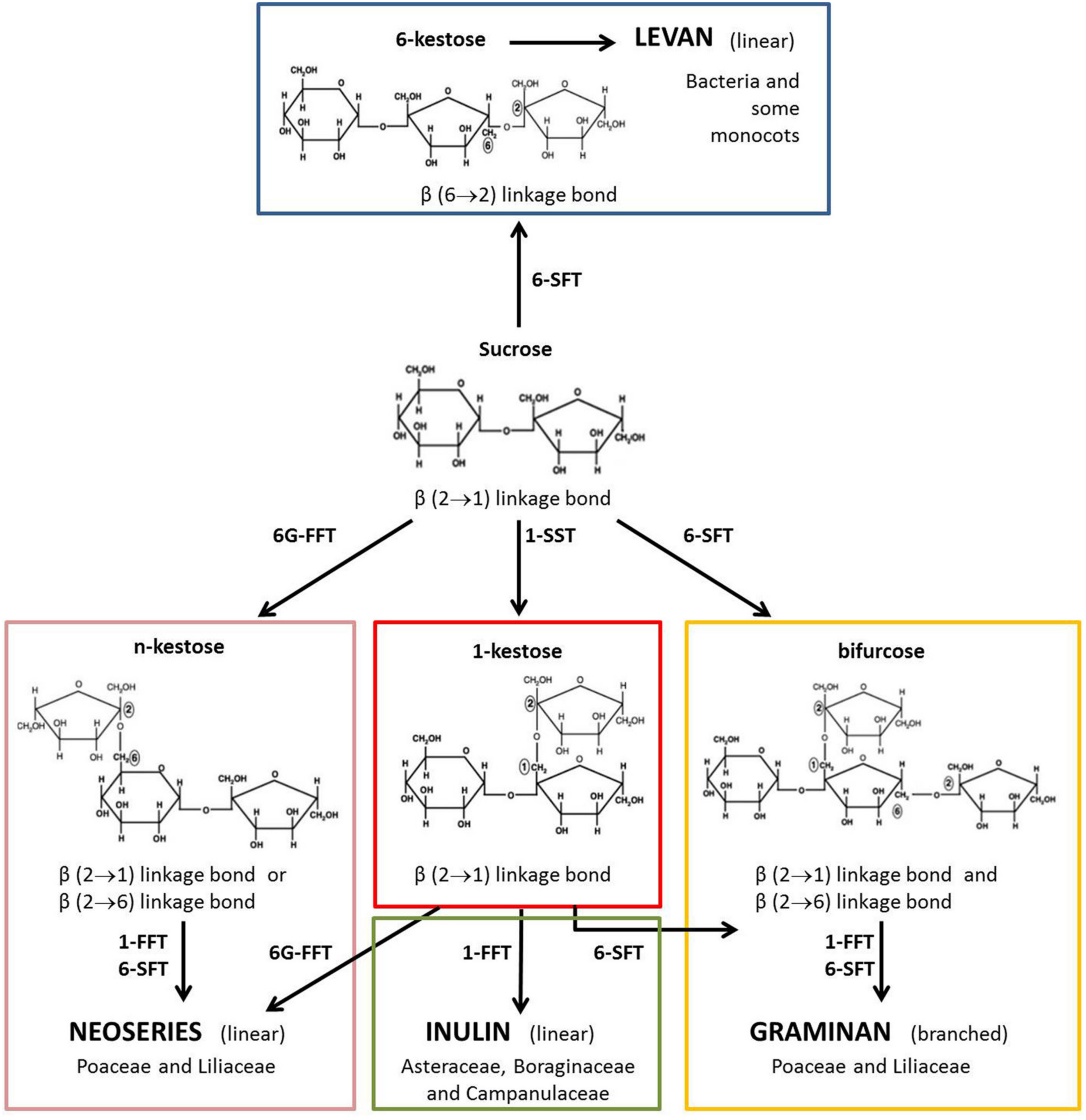
**Model for fructan biosynthesis. Fructans are synthesized starting from Suc**. They are linear or branched polysaccharides. In higher plants, fructans are classified into four structurally distinct major categories depending on the position of the glucosyl unit and on the type of glycosidic linkage between fructosyl residues: inulin, levan, graminan and neoseries fructan can be discerned (Ritsema and Smeekens, 2003). Fructan biosynthesis is mediated by several fructosyltransferases: 1-SST (sucrose:sucrose 1-fructosyltransferase); 1-FFT (fructan:fructan 1-fructosyltransferase); 6-SFT (sucrose:fructan 6-fructosyltransferase) and 6G-FFT (fructan:fructan 6G-fructosyltransferase).

Cereal kernel fructans are predominantly of the graminan-type, i.e. branched molecules characterized by β(2–1) and β(2–6) linkages between the fructosyl residues and with a terminal Glc residue. However, neoseries fructans, which have an internal Glc residue, have also been found in cereals (Nilsson and Dahlqvist, 1986; Verspreet et al., 2013a,b). Fructan metabolism is mediated by a complex set of biosynthetic and hydrolytic enzymes (Figure 1). Enzymes involved in fructan biosynthesis have been identified in the vegetative tissues of wheat: sucrose:sucrose 1-fructosyltransferase (1-SST), fructan:fructan 1-fructosyltransferase (1-FFT) and sucrose:fructan 6-fructosyltransferase (6-SFT) (Yoshida et al., 2007; Gao et al., 2010). Fructan degradation is catalyzed by fructan exohydrolases (FEH). In wheat, three FEH types have been detected: 1-FEH, 6-FEH and 6&1-FEH (Van den Ende et al., 2003, 2005; Van Riet et al., 2006, 2008).

In order to better understand fructan metabolism during durum wheat kernel maturation and due to the nutritional and health interests in fructans (Van den Ende et al., 2011a; Pasqualetti et al., 2014), fructan profile and content and the activity as well as gene expression of the known fructan metabolizing enzymes were studied during kernel development.

## Material and methods

### Plant material

Plants of *Triticum durum* Desf. (cv Neolatino) were grown in experimental fields in Rome in 2010–2011 on 10 m^2^ plots with a sowing density of up to 450 seeds/m^2^. The plants were arranged in a randomized block design, and collected from two segments of a 25 cm length in the same row. Irrigation, fertilization and plant protection were performed to ensure optimal plant growth. After flowering, ears were collected weekly from 7 to 52 DAA (complete kernel development). The ears were separated from the stems and stored at −80°C. Kernels collected from the middle part of the ears were ground in liquid nitrogen and immediately used or dehydrated by lyophilisation and stored at −20°C.

### Starch content

Starch content was analyzed using the AOAC 996.11/AACC 76.13 Method. Megazyme's Total Starch kit (K-TSTA 07/11, Megazyme International Ireland Ltd, Bray, Ireland) was used following the manufacturer's instructions.

### Carbohydrate extraction

To denature enzymes, 50 mg of durum wheat kernel ground in liquid nitrogen were heated at 90°C in 1 ml ethanol, until all ethanol evaporated. Rhamnose (0.25 mg/ml) was used added as an internal standard. Subsequently, a water extraction occurred in a shaking water bath at 80°C for 1 h. After incubation and cooling, the sample was centrifuged at 9000 g for 10 min at room temperature. A solution of rhamnose, melibiose, Glc, Fru and Suc with a concentration of 5 μg/ml was used as calibration solution.

### Carbohydrate measurements by HPAEC-PAD

Carbohydrates were analyzed with high performance anion exchange chromatography with pulsed amperometric detection (HPAEC-PAD), performed on a Dionex ICS 3000 chromatography system (Sunnyvale, CA, USA). Analysis and detection were performed at 32°C and the flow rate was 250 μl per min. A 15 μl sample was injected on a Guard CarboPac PA 100 (2 × 50 mm) in series with an analytical CarboPac PA 100 (2 × 250 mm) equilibrated for 9 min with 90 mM CO_2_-free NaOH. Sugars were eluted in 90 mM NaOH, with an increasing sodium acetate gradient: from 0 to 6 min, the sodium acetate concentration increased linearly from 0 to 10 mM; from 6 to 16 min from 10 to 100 mM; and from 16 to 26 min from 100 to 175 mM. The columns were then regenerated with 500 mM sodium acetate for 1 min and equilibrated with 90 mM NaOH for 9 min for the next run. Data were recorded and processed with Chromeleon software. In order to determine the total fructan content, 2.5 μl of 1.2 M HCl-solution was added to 50 μl of the watery extract (see above) and incubated for 90 min at 70°C. The hydrolysis was stopped by adding 2 μl of 1 M H_2_CO_3_. Deionized water was added up to a final volume of 1 ml, and the mixture was analyzed on a HPAEC-PAD. The fructan concentration and DP were calculated as described in Verspreet et al. (2012).

### Determination of enzyme activities

To determine the activities of fructan enzymes, 50 mg of freeze-dried wheat kernel samples were homogenized in 600 μl of 50 mM sodium acetate pH 5.0 containing 1 mM β-mercaptoethanol, 10 mM sodium bisulfite, 0.1% (w/v) polyclar and 0.02% (w/v) sodium azide, and 3 μl of 200 mM phenylmethylsulfonyl fluoride dissolved in pure ethanol. The enzymes were precipitated using solid ammonium sulfate (80% saturation) and suspended in sodium acetate buffer pH 5.0 containing 0.02% sodium azide. Proteins were measured according to Bradford (1976), using bovine serum albumin as a standard.

The substrates used for measuring enzyme activities were suspended in 50 mM sodium acetate buffer pH 5.0 with 0.02% sodium azide. 1-Kestotriose (1-K) was purchased from Sigma Aldrich. 6^G^-kestotriose, also termed neokestose (n-K) was purified from *Xanthophyllomyces dendrorhous* culture broth as described (Kritzinger et al., 2003). Kernel substrate (KS) is a carbohydrate extract from *T. aestivum* immature kernels without hexoses. The reaction mixtures containing 20 μl of enzyme extract were incubated with 50 mM sodium acetate buffer pH 5.0 with a single or a mix of the following substrates: Suc, n-K, 1-K, KS. All the substrates were used at the concentration of 50 mM, with the exception of Suc which was added at 200 mM concentration when used as a single substrate, and KS which were used at 2 mM. The reaction was carried out at 30°C and stopped at 95°C for 5 min. Samples were analyzed by HPAEC-PAD, as previously described (Verspreet et al., 2012).

### Gene expression analyses

The RNA was extracted from *T. durum* kernels collected at 7, 14, 21, 28, 35, and 52 DAA and grown in liquid nitrogen. Total RNA was extracted by TRIzol® Reagent (Ambion Waltham, MA USA; 15596-018) following the manufacturer's instructions. Plant RNA Isolation AID (Ambion Waltham, MA USA; AM9690) was used to facilitate the removal of polysaccharides and polyphenols. RNA purity was estimated by measuring 260/280 and 260/230 wavelength ratio. Denaturing gel electrophoresis was used to visually assess the quality of RNA. DNase treatment was performed using TURBO DNA-free Kit (Applied Biosystems, Waltham, MA USA; AM1907). RNA was reverse transcribed using High Capacity RNA-to-cDNA Kit (Applied Biosystems, Waltham, MA USA; 4387406) following the manufacturer's instructions. Specific primers were designed for the 18S gene and for the genes involved in fructan metabolism, using the software Primer 3 available on-line http://frodo.wi.mit.edu/, and synthesized by Primm (Milan, Italy).

The polymerase chain reaction (PCR) was carried out using the Advantage-GC cDNA Polymerase Mix (Clontech Laboratories, Inc., Mountain View, CA, USA; 639112), according to the manufacturer's instructions. Supplementary Table 1 lists the specific primers used for the PCR reactions, the optimal primer annealing temperature and the number of cycles required in order to reach the PCR exponential phase. Images of EtBr-stained agarose gels were acquired with a ChemiDoc™ XRS (Bio-Rad Laboratories, Inc., Hercules, CA, USA). Band quantification was performed by Image Lab™ software (Bio-Rad Laboratories, Inc.). Band intensity was expressed as relative absorbance units. Normalization with respect to a positive control 18S was calculated to normalize variations in sample concentration and as a control for reaction efficiency.

### Statistics

The values obtained for metabolites and RT-PCR were the mean of three independent experiments ± SD. Enzyme activities were performed in two independent experiments ± SD. Where indicated, an ANOVA test was used to verify the statistical significances among the different values obtained during kernel maturation.

## Results

Kernel development of *T. durum* cv. Neolatino was studied from 7 DAA to physiological maturation (52 DAA, see Material and Methods for details). At 12–15 DAA the kernels were at the milky phase, while the dehydration process started at 28 DAA (data not shown).

### Carbohydrate contents

**Figure 2:**
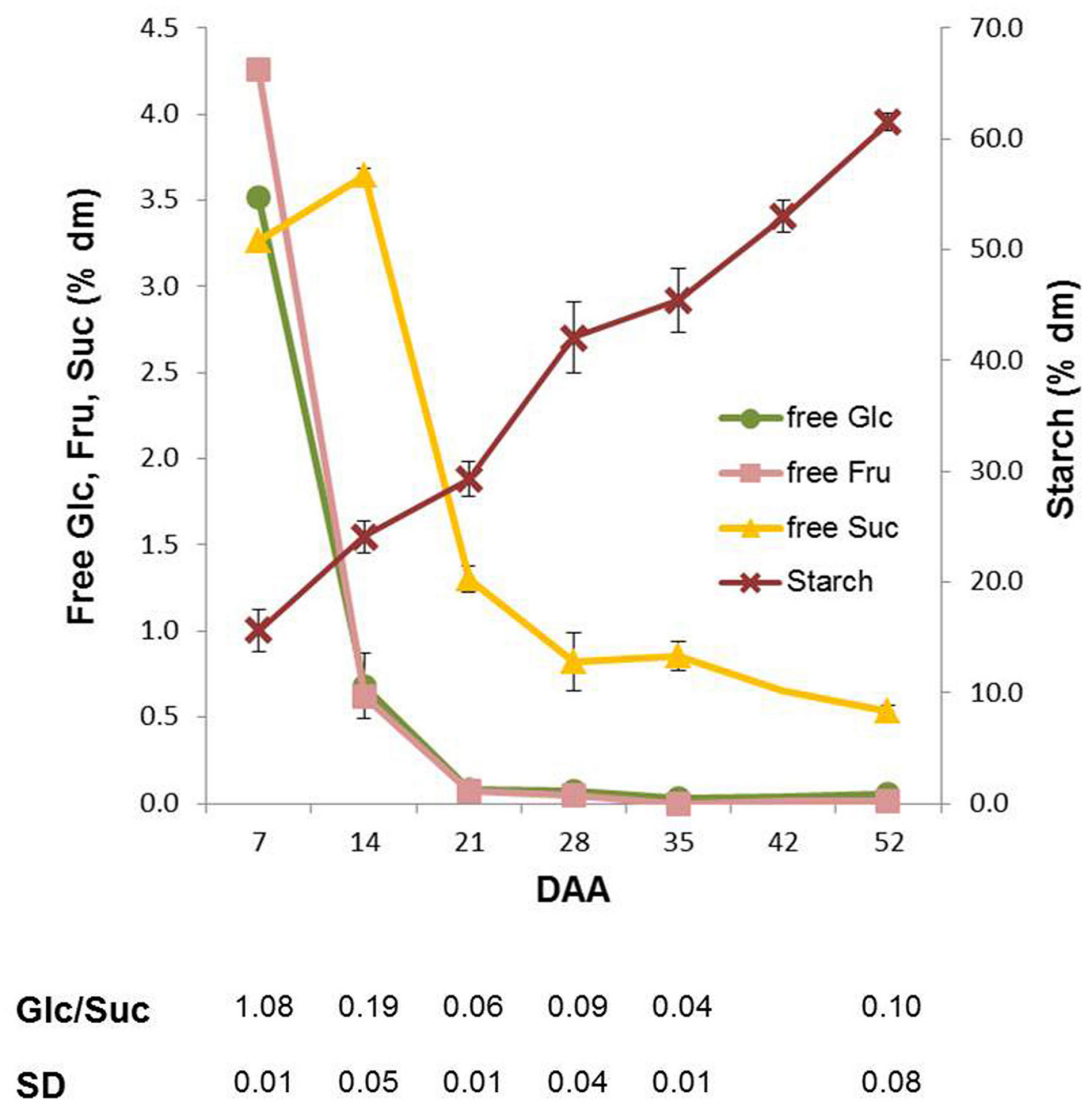
**Glucose (Glc), fructose (Fru), sucrose (Suc) and starch content in Neolatino kernels collected from 7 and 52 DAA**. All the values are expressed as % dm.

Variations in the content of the main carbohydrates in wheat kernels were analyzed during the whole kernel maturation period. The highest amounts of Fru and Suc were observed at 7 DAA. The contents of Fru and Glc, representing 4% of dm at 7 DAA, decreased rapidly between 7 and 14 DAA (more than 90%), and a further decrease occurred in the following maturation period. At physiological maturation, kernels contained very low amounts of these monosaccharides (Figure 2). The content of sucrose (Suc) also decreased during maturation, but followed a different trend: it transiently increased until 14 DAA (from 3 to 3.6% of dm; *P* < 0.01 by ANOVA test), then it decreased until the end of maturation when the level of Suc was only 0.5% of dm (Figure 2). As a consequence, the Glc/Suc ratio considerably changed during kernel maturation. As expected, the total starch content increased during kernel maturation from 12–15% of dry matter (7 DAA) to about 60% at the end of maturation process (Figure 2).

**Figure 3:**
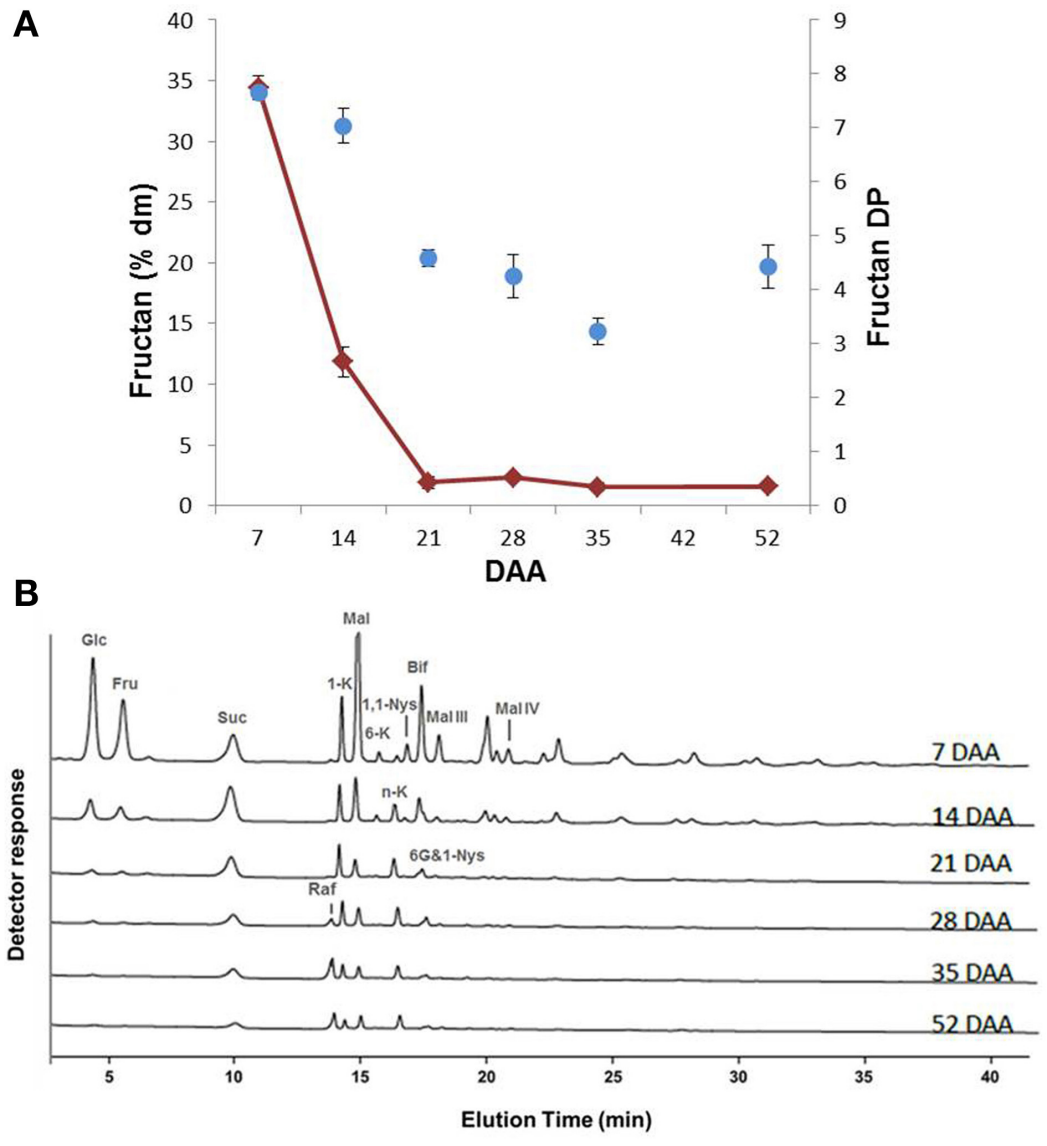
**(A) Fructan content of durum wheat kernels at different DAA**. The values are expressed as % dm. Changes of fructan DP during durum wheat kernel maturation. **(B)** Qualitative sugar profiles of durum wheat kernels at different DAA performed by HPAEC-PAD. Known compounds are indicated: glucose (Glc), fructose (Fru), sucrose (Suc), 1-kestotriose (1-K), maltose (Mal), 6-kestotriose (6-K), neokestose (n-K), 1,1-nystose (1,1-Nys), bifurcose (Bif), 1&6G-kestotetraose (6G&1-Nys), raffinose (Raf), maltotriose (Mal-III) and maltotetraose (Mal-IV).

The variation in total fructan content showed a similar trend as the monosaccharides, with the highest values at 7 DAA (35% of dm) and a progressive decrease until 21 DAA, after which the fructan level remained at an approximately constant value of 2–3% of dm (Figure 3A). An analysis of the average degree of polymerization (DP) of fructans revealed that the average DP lowered during durum wheat kernel maturation (Figure 3A).

The HPAEC-PAD profiles during the various phases of kernel maturation indicated qualitative variations in fructan contents during kernel maturation (Figure 3B). Apart from Glc, Fru and Suc, maltose (Mal) was the most abundant sugar found during the early phase of kernel maturation (7 DAA) (Figure 3B). At this stage, the chromatographic profile revealed the presence of several fructans, including 1-kestotriose (1-K), 6-kestotriose (6-K), 6G-kestotriose or neokestose (n-K), 1,1-nystose (1,1-Nys) and bifurcose or 1&6-kestotetraose (Bif) (or other co-eluting tetrasaccharides). Maltotriose (Mal-III) and Maltotetraose (Mal-IV) were also identified. During the following phases of maturation, most of the molecules identified at 7 DAA decreased or disappeared; only n-K remained almost constant, while 1&6G-kestotetraose (6G&1-Nys) persisted longer (Figure 3B). Raffinose (Raf) increased starting from 28 DAA (Figure 3B).

### Fructan metabolism

In order to evaluate the variations in the activities of soluble fructan and Suc metabolizing enzymes, protein extracts from kernels collected at different developmental stages were incubated with different substrates. The products obtained were identified by HPAEC-PAD, as described above.

**Figure 4:**
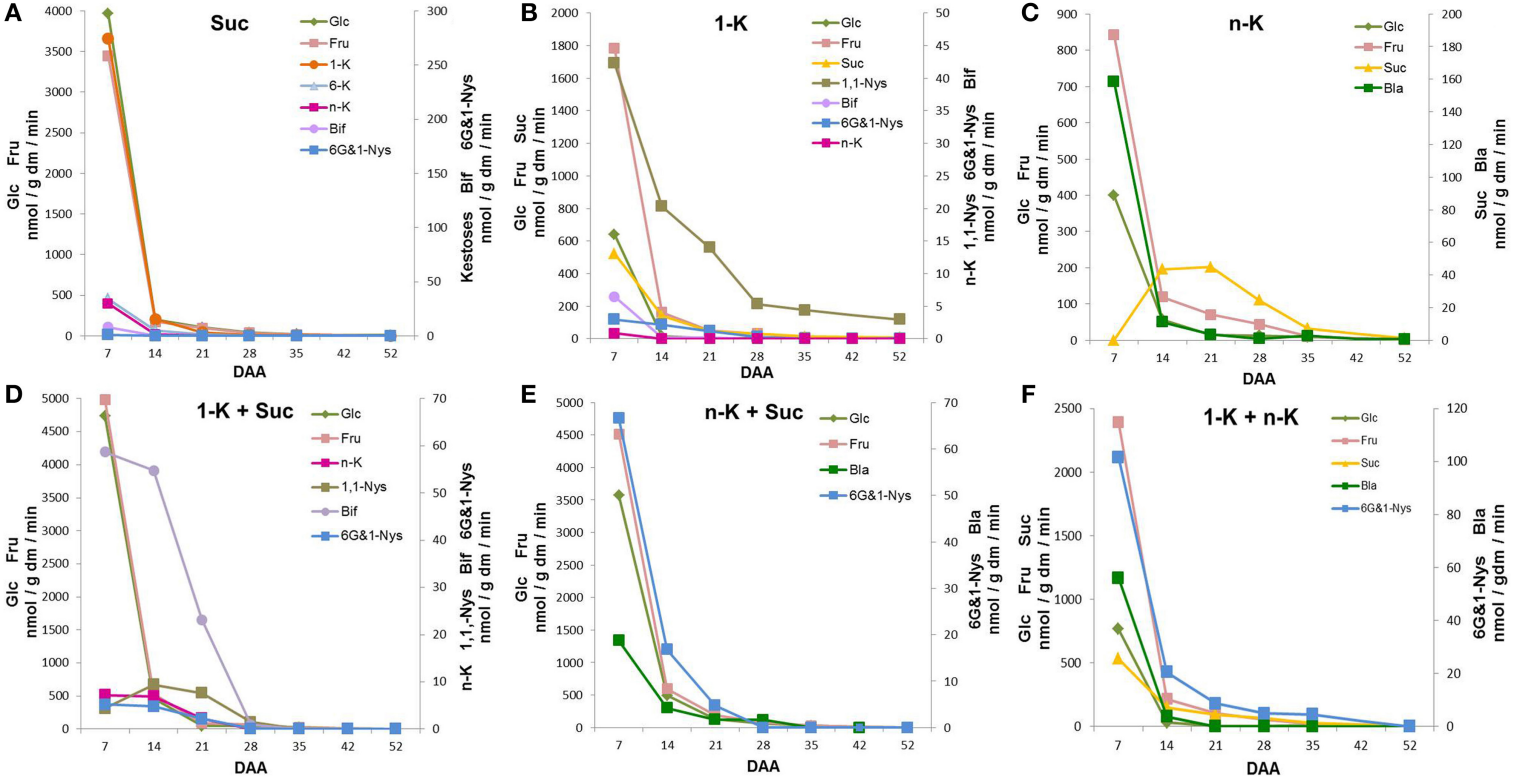
**Sugars produced after incubation of kernel enzymatic extracts with (A) sucrose (Suc) as a single substrate; (B) 1-kestotriose (1-K) as single substrate; (C) neokestose (n-K) as a single substrate; (D) a combination of 1-K and n-K as substrates; (E) a combination of 1-K and Suc as substrates; (F) a combination of n-K and Suc as substrates**. All the values are expressed as nmol/g dm/min.

Figure 4A shows that 7 DAA kernel protein extracts incubated with Suc led to abundant Fru and Glc formation, demonstrating that a strong soluble acid invertase activity was present in immature kernels. The biosynthesis of fructans with DP3, namely 1-K, 6-K, and n-K, was also observed at 7 DAA. In addition, a small amount of the tetra-oligosaccharides 6G&1-Nys and Bif were detectable at 7 DAA. During the subsequent 2 weeks of kernel maturation, all these metabolites remained present in similar proportions, but their amounts decreased until almost undetectable (Figure 4A).

When kernel protein extracts were incubated with 1-K, the synthesis of DP4 fructans occurred (Figure 4B). In addition to 1,1-Nys, small amounts of 6G&1-Nys and Bif were produced. The 1,1-Nys synthesis was greater and lasted for longer than the 6G&1-Nys synthesis, probably produced by 1-FFT and 6G-FFT, respectively. High 1-K degradation was also detected at 7 DAA, as confirmed by Glc, Fru and Suc production (Figure 4B). When n-K was supplied as a single substrate, no synthesis of oligosaccharides with higher DP was observed (Figure 4C).

Only the monosaccharides Glc and Fru and the disaccharide blastose (Bla), a breakdown product of n-K after the release of one Fru moiety by invertase action, were observed at 7 DAA. In the presence of n-K as substrate, Suc accumulation transiently increased reaching its maximum between 14 and 21 DAA, after which it progressively decreased during the last period of kernel maturation (Figure 4C). On the other hand, there was a 14-fold decrease in Bla production between 7 and 14 DAA, a further decrease occurred from 14 to 21 DAA, after which low levels were produced until the end of kernel maturation (Figure 4C).

When 1-K plus Suc were combined as substrates, the synthesis of the tetra-saccharide Bif occurred (Figure 4D), demonstrating the presence of 6-SFT activity. This enzyme uses Suc as donor, and 1-K as preferential acceptor substrate of fructosyl units. The maximum amount of Bif was produced at 7 DAA, after which Bif biosynthetic capability decreased until almost undetectable values at 28 DAA. In addition, a small amount of n-K, as a consequence of the activity of 6G-FFT, was observed from 7 DAA until 14 DAA, after this stage of maturation n-K production was also almost undetectable. Under these conditions, 1,1-Nys synthesis was also observed. 1,1-Nys transiently increased from 7 to 14 DAA and then decreased reaching very low levels (Figure 4D). Finally, a low amount of 6G&1-Nys was produced at 7 and 14 DAA (Figure 4D).

Kernel enzymatic extracts were also incubated with n-K and Suc (Figure 4E). Under these conditions, the major tetrasaccharide formed was 6G&1-Nys, suggesting that Suc acted as a donor substrate and n-K as an acceptor substrate in a reaction catalyzed by a putative sucrose:fructan 1-fructosyltransferase or 1-SFT. The synthesis of 6G&1-Nys showed its maximum value at 7 DAA, after which it rapidly decreased. A small amount of Bla was also detectable (Figure 4E). However, Bla production was 30 times higher (at 7 DAA) when n-K was used as the only substrate (Figure 4C). Invertase-mediated breakdown of Suc also occurred (Figure 4E).

When a combination of 1-K and n-K was used as substrate, the enzyme 1-FFT catalyzed the synthesis of 6G&1-Nys and Suc from 1-K as a donor substrate and n-K as an acceptor substrate (Figure 4F). Part of the Suc produced was then hydrolyzed to Glc and Fru. Most of the 6G&1-Nys production occurred at the highest rate at 7 DAA, it dropped sharply in the following seven days (80%), after which it slowly decreased and became undetectable at 52 DAA. Bla and an additional amount of Fru were produced from n-K breakdown catalyzed by invertase (Figure 4F). However, under these conditions, Bla was formed at a lower rate as compared with n-K as a single substrate (Figure 4C).

**Figure 5:**
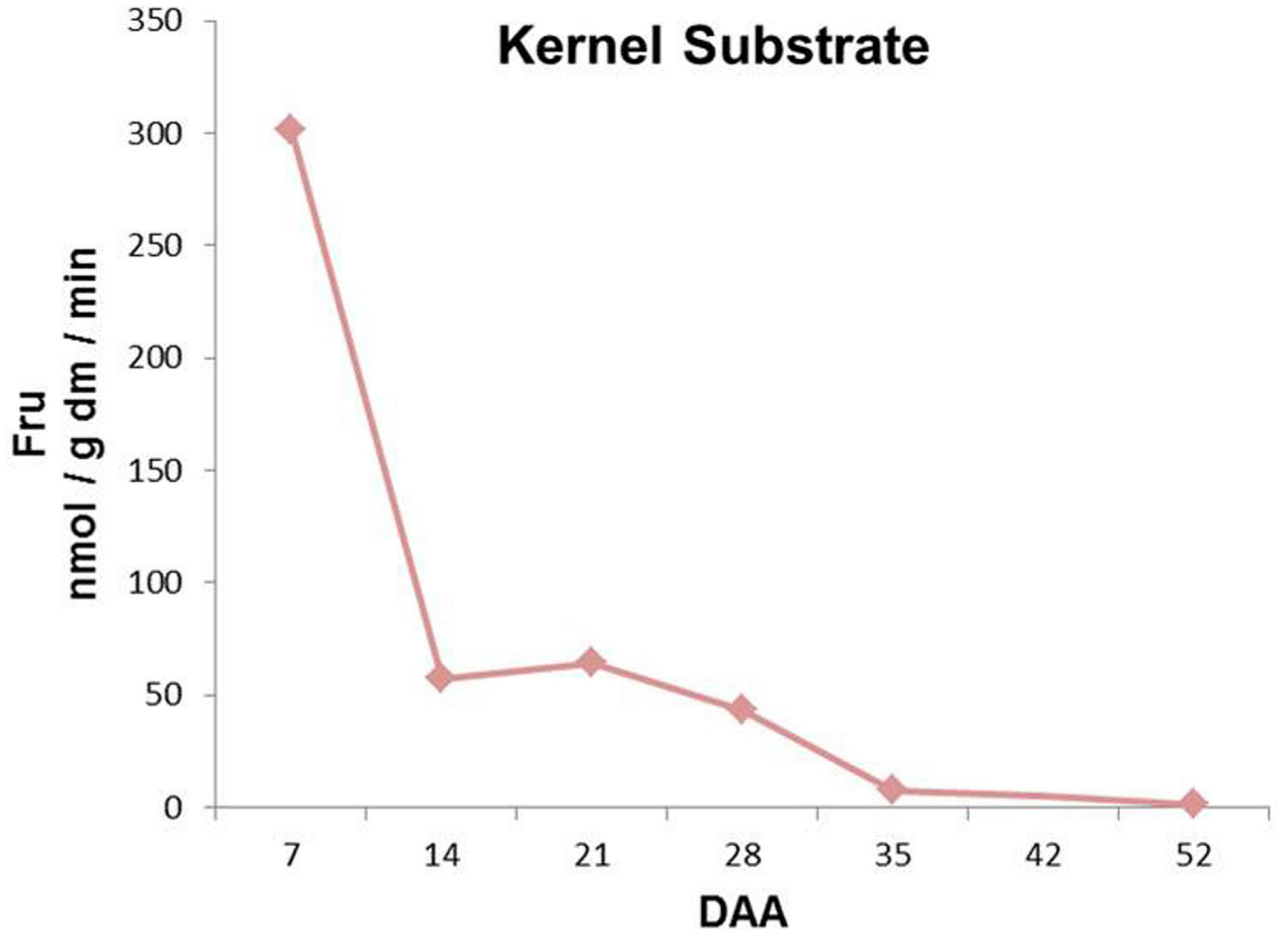
**Sugars produced after incubation of kernel enzymatic extracts with wheat kernel graminans and neoseries fructans as substrates at different stages of kernel maturation**. All the values are expressed as nmol/g dm/min.

Kernel enzymatic extracts with endogenous kernel-derived fructans were incubated to evaluate total FEH activities during kernel development (Figure 5). Fructan breakdown capacity peaked at 7 DAA, after which it gradually decreased until the end of kernel maturation (Figure 5).

**Figure 6:**
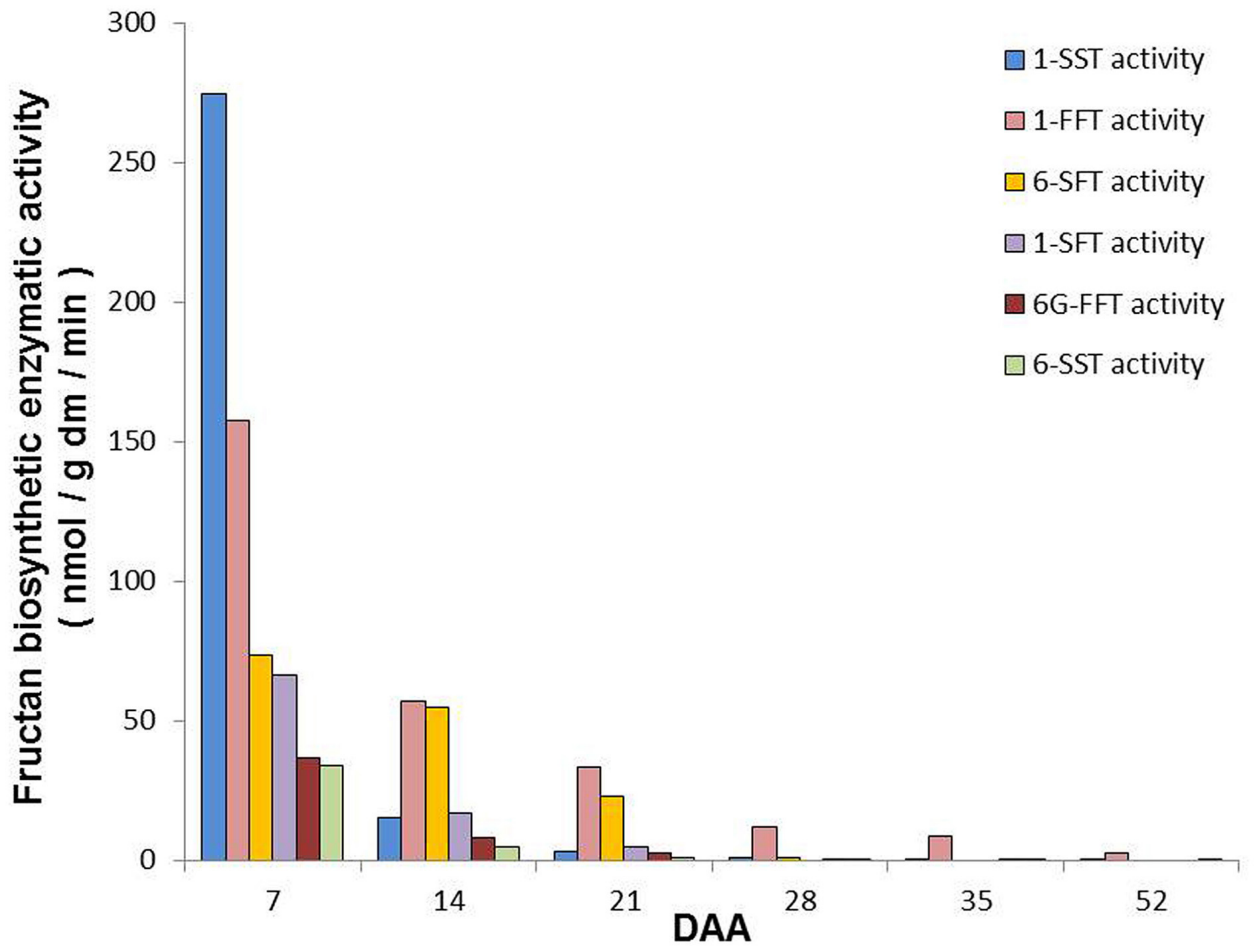
**Summary of the main fructan biosynthetic activities observed in durum wheat kernels during maturation**. The values are expressed as nmol/g dm/min and have been calculated on the basis of main activities detected by using different substrates (see Figure 4) according to Figure 1.

Figure 6 summarizes the main biosynthetic activities observed in *T. durum* kernels during maturation. Even though the activity of all the identified enzymes was higher at 7 DAA than in the following periods, they decreased at different rates during kernel maturation.

### Fructan gene expression

**Figure 7:**
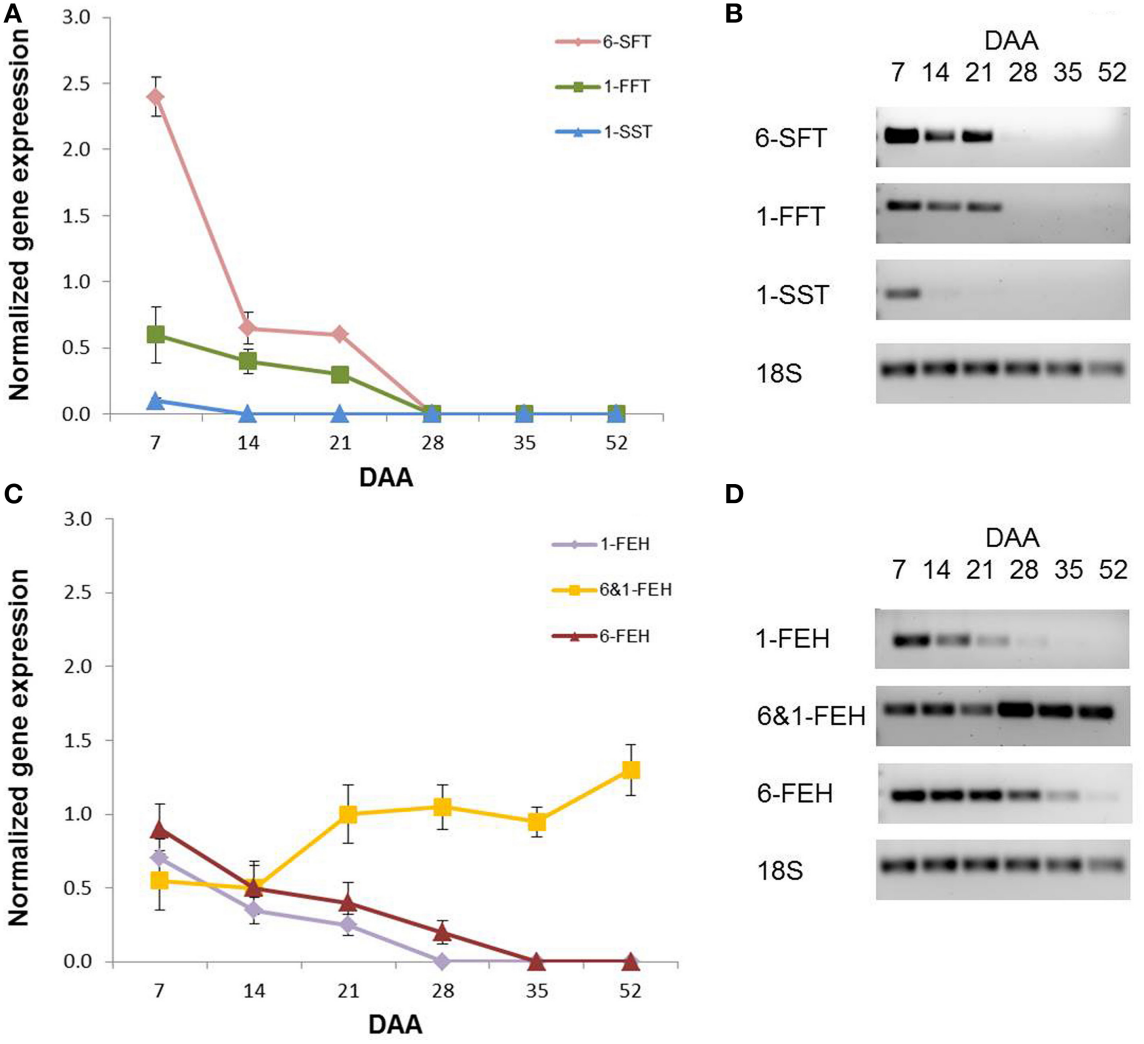
**Semi-quantitative gene expression of (A) 6-SFT, 1-FFT, 1-SST and (C) 1-FEH, 6&1-FEH and 6-FEH in wheat kernels at 7, 14, 21, 28, 35, and 52 DAA. (B)** representative semi quantitative PCR gel images of 6-SFT, 1-FFT, 1-SST, 18S. **(D)** representative semi quantitative PCR gel images of 1-FEH, 6&1-FEH, 6-FEH, 18S. Values were normalized using 18S gene expression as the housekeeping.

A semi-quantitative PCR analysis was performed on the fructan genes that had been already cloned from wheat, namely *1-SST, 1-FFT, 6-SFT, 1-FEH, 6-FEH* and *6*&*1-FEH* (Supplementary Table 1). As with the enzymatic activities, during kernel development there was a progressive decrease in the expression of all fructan biosynthetic genes (Figure 7A). The expression of 1-SST encoding gene was already undetectable at 14 DAA, while the gene expression of 6-SFT and 1-FFT became undetectable at 28 DAA (Figure 7A). Considering the expression of FEH genes, different behaviors were observed for 1-FEH and 6-FEH versus 6&1-FEH. The gene expression of 1-FEH and 6-FEH decreased progressively during development, reaching undetectable levels at 28 and 35 DAA respectively (Figure 7B). By contrast, the transcript levels of the 6&1-FEH gene were constant between 7 and 14 DAA. It increased at 21 DAA, after which it remained constant until physiological maturation (Figure 7B; *P* < 0.05 by Anova test between the values of 7 and 14 DAA versus the values of the following DAA).

## Discussion

Kernel development is a complex process where the fluxes of metabolites are first involved in cell proliferation and differentiation, and then in the storage of macromolecules and nutrients. Several partners are involved in the regulation of the process: from carbohydrate availability to hormone and sugar signaling pathways. Molecules involved in redox homeostasis also play a key role in controlling these processes (De Gara et al., 2003; Paradiso et al., 2012). Interestingly, the biosynthesis of metabolites involved in redox homeostasis, such as ascorbate, strictly depend on the regulation of sugar metabolic fluxes (Locato et al., 2013).

Suc is the main source of sugars for kernel metabolism mainly through the phloematic flux from source organs, though some carbohydrates are also synthesized by the photosynthetically active tissues of the kernel (Rolletschek et al., 2004). We found that the Suc level increased until 14 DAA after which it decreased, while Glc and Fru had the highest levels at the very beginning of kernel maturation (Figure 2).

In kernels, a high Glc/Suc ratio characterizes the phase of endosperm cell proliferation, while a spike in Suc concentration marks the transition into the starch accumulation phase (Sabelli and Larkins, 2009). In line with Sabelli and Larkins we found a high Glc/Suc ratio at 7 DAA and a spike in Suc that preceded the phase of more intense starch accumulation (Figure 2). In immature kernels, fructan levels showed values about 10 times higher than those of Suc, Fru, Glc, representing 35% and 10% of dry matter at 7 and 14 DAA, respectively (Figures 2, 3). Some fructans (mainly in the form of tri- and tetrasaccharides) were still present in the mature kernels (2–3% of dm), while Suc was only 0.5 % of dm, and Fru and Glc were almost undetectable starting from 35 DAA (Figures 2, 3).

During kernel maturation Raf is stored starting from 28 DAA, at the beginning of kernel dehydration, in line with its role in the acquisition of desiccation tolerance (Bailly et al., 2001; Van den Ende, 2013).

During early development, using Suc as the primary biosynthetic precursor, fructan production from Suc may allow green tissues of immature kernels to prevent negative feedback on photosynthesis (Pollock, 1986; Koroleva et al., 1998). Indeed the highest amount and DP of fructans observed early in kernel development (Figure 3) supports the idea that fructan biosynthesis preceding the massive starch accumulation can also contribute to make kernels more effective Suc sinks, since Suc is promptly used for fructan polymerization. The need to metabolize Suc in the first phases of kernel maturation is also supported by the invertase activity. In fact, the highest soluble, acid invertase activity was recorded at 7 DAA in durum wheat kernels. FEH-mediated fructan degradation may lower the osmotic potential mediating drought resistance *in planta* (De Roover et al., 2000; Livingston et al., 2009). Therefore, fructan metabolism may also participate in osmotic regulation during kernel development.

Fructan and Suc metabolisms may play complex roles in kernel maturation, also linked to sugar signaling. In fact the fructosyl transferase enzymes that catalyze the various reactions of fructan elongation and branching are multifunctional enzymes, which are able to catalyze different reactions depending on the substrate availability (Van den Ende et al., 2009, 2011b). In this study, the activities of fructan enzymes were measured by incubating kernel extracts with high levels of their substrates; such conditions may not necessarily reflect the exact *in vivo* cellular environment occurring during the different phases of kernel maturation. Despite this, the picture emerging from the analysis of the enzymes of fructan biosynthesis concurs with their levels during kernel development. The activity of these enzymes decreased throughout the period analyzed (Figure 6).

In line with fructan levels, all the assayed biosynthetic enzymatic activities showed their highest values at the early developmental stage, with 1-SST having the highest activity at 7 DAA (Figure 6). The activity of this enzyme decreased more rapidly than the other biosynthetic enzymes, since at 14 and 21 DAA 1-FFT and 6-SFT had a higher activity than 1-SST, which is considered to be the key enzyme for starting fructan biosynthesis in general (Ritsema and Smeekens, 2003) and in wheat in particular (Housley and Daughtry, 1987). Interestingly the activities of the two enzymes mainly involved in graminan biosynthesis (1-FFT and 6-SFT; see Figure 1) were maintained longer compared to those of the other biosynthetic enzymes (Figure 6). The transcript levels of fructan biosynthesis genes in this study also decreased during kernel maturation, with 6-SFT and 1-FFT mRNAs retaining longer than those of 1-SST (Figures 6, 7A). The expression of the genes encoding the enzymes of fructan breakdown also decreased during kernel maturation (Figure 7B).

The high activity of FEHs observed at the beginning of the developmental phase could be explained by the trimming feature attributed to 1-FEH (Bancal et al., 1992), which is likely to be responsible for the greatest level of complexity of the fructan pool observed at the beginning of kernel development, as reported in the chromatogram of sugars obtained from kernels at 7 DAA in Figure 3B. However, an alternative explanation is that the high 1-FEH activities at 7 DAA (Figure 4B) could be considered as an intrinsic property of the overwhelming vacuolar invertase activity at 7 DAA, as demonstrated before for rice vacuolar invertases (Ji et al., 2007). Unlike 6&1-FEH transcripts, 1-FEH and 6-FEH mRNAs progressively decreased during kernel maturation, reaching negligible levels at 35 DAA (Figure 7B).

The capability of wheat kernels to retain a certain amount of fructans at the end of their developmental stage might be correlated with the feature of fructans to stabilize membranes. The relevance of fructans in stabilizing membranes has been underlined under thermic and drought stress (Livingston et al., 2009) and might also play a role in fastening and optimizing the membrane reorganization during kernel hydration in the first phases of kernel germination. Moreover, fructan breakdown seems to be less expensive in terms of ATP consumption than starch breakdown (Kötting et al., 2005), thus retaining a certain amount of fructans in mature kernels could represent a metabolic advantage during the early stage of germination. Supporting this hypothesis, mature kernels still contain fructans and the mRNA encoding for the fructan exohydrolase 6&1-FEH (Figure 7B), though further evidence is required in order to verify the relevance of rest fructan as source of carbohydrates and in stabilizing membranes during subsequent kernel germination.

Fructans are not only a source of carbon and energy, but are also compounds involved in stress responses (Livingston et al., 2009), perhaps even acting as signals (Van den Ende, 2013; Peshev and Van den Ende, 2014). Moreover, emerging data suggest that fructans might fulfill a significant role as localized ROS scavengers both in plants and in human health (Stoyanova et al., 2011; Peshev et al., 2013; Pasqualetti et al., 2014; Peshev and Van den Ende, 2014). Therefore, their increase might contribute to the regulatory mechanisms controlling the early wheat development stages, while their decrease may mark the transition to later developmental stages, orchestrated by the changes in redox balance during these processes.

In conclusion, our results suggest that the quali/quantitative variations in fructan pool during kernel maturation might be part of the signaling pathways regulating carbohydrate metabolism and storage in wheat kernels, as well as fructan retaining in mature kernels might improve germination efficiency.

In spite of the low yield obtainable at milky stage the very high levels of fructans in the kernels collected at this stage are also noteworthy for the production of functional foods in which immature kernels are the source of bioactive molecules.

### Conflict of interest statement

The authors declare that the research was conducted in the absence of any commercial or financial relationships that could be construed as a potential conflict of interest.
