# Supplementary material for: Fructan biosynthesis and degradation as part of plant metabolism controlling sugar fluxes during durum wheat kernel maturation
Source: Front Plant Sci. 2015 Feb 20;6:89. doi: 10.3389/fpls.2015.00089 (PMC4335405; doi:10.3389/fpls.2015.00089)
Supplement: Supplementary file 1 [file Table1.DOCX]

***Supplementary Material***

**Fructan biosynthesis and degradation as part of plant metabolism controlling sugar fluxes during durum wheat kernel maturation.**

Sara Cimini^1°^, Vittoria Locato^1°^, Rudy Vergauwen^2°^, Annalisa Paradiso^3^, Cristina Cecchini^4^, Liesbeth Vandenpoel ^2,1^, Joran Verspreet^5^, Christophe M. Courtin^5^, Maria G. D’Egidio^4^, Wim Van den Ende^2*^, Laura De Gara^1*^

°equally contributing authors

*corresponding authors

^1^Laboratory of Plant Biochemistry and Food Sciences, Campus Bio-Medico University, Rome, Italy

^2^Laboratory for Molecular Plant Biology and Leuven Food Science and Nutrition Research Centre (LFoRCe), KU Leuven, Leuven, Belgium

^3^Dipartimento di Biologia, Università degli Studi di Bari, Bari, Italy

^4^Consiglio per la Ricerca e la Sperimentazione in Agricoltura, Unità di ricerca per la Valorizzazione Qualitativa dei Cereali, Rome, Italy

^5^Laboratory of Food Chemistry and Biochemistry, KU Leuven, Leuven, Belgium

*** Correspondence:**

Laura De Gara, Laboratory of Plant Biochemistry and Food Sciences, Campus Bio-Medico University, Via Alvaro del Portillo 21, Rome, 00128, Italy.

[l.degara@unicampus.it](mailto:l.degara@unicampus.it)

Wim Van den Ende, Laboratory for Molecular Plant Biology and Leuven Food Science and Nutrition Research Centre (LFoRCe), KU Leuven, Kasteelpark Arenberg 31, Leuven, B-3001, Belgium

[wim.vandenende@bio.kuleuven.be](mailto:wim.vandenende@bio.kuleuven.be)

1. **Supplementary Figures and Tables**

## Supplementary Tables

**Supplementary Table 1.** Primer sequences and reaction conditions used for semi-quantitative RT-PCR are reported.

| Gene Name | Accession Number | Primer Sequence | T annealing (°C) | N° of cycles |
| --- | --- | --- | --- | --- |
| 18S | AY049040.1 | FW 5’-GAGCCTGCGCTTAATTTGAC-3’  Rev 5’-TAGCAGGCTGAGGTCTCGTT-3 | 52 | 24 |
| 1-FEH | AJ564996.1 | FW 5’-ACTGGTGGTGACATAGATCAAA-3’  Rev 5’-CTGTAGCCGTTCAGCTCAC-3’ | 52 | 30 |
| 6&1-FEH | AB089269.1 | FW 5’-CCCAGTGATCCAACATGTCA-3’  Rev 5’-GCGTTCAATTCTAGTCCAACTCAT-3’ | 54 | 34 |
| 6-FEH | AM075205.1 | FW 5’-CGACTATAATGGCTGTTGGTCA-3’  Rev 5’-GTCGGCTGGGAGTGGTATG-3’ | 56 | 36 |
| 6-SFT | AB029887.1 | FW 5’-AGTTCCAAGGACAATTGCTCTC-3’  Rev 5’-ACGGCAGAAGCATCAAGGT-3’ | 54 | 32 |
| 1-FFT | AB088409.1 | FW 5’-GGTGGGGCAACATCTCATG-3’  Rev 5’-CGTGGTTGGGTCACGGTAG-3’ | 54 | 36 |
| 1-SST | AB029888.1 | FW 5’-CGTGTCCAAGGACTTCATTC-3’  Rev 5’-GCCGTCGGACTCGTCGTA-3’ | 54 | 40 |
